# Supplementary material for: Spectroscopy of bulk and few-layer superconducting NbSe2 with van der Waals tunnel junctions
Source: Nat Commun. 2018 Feb 9;9:598. doi: 10.1038/s41467-018-03000-w (PMC5807409; doi:10.1038/s41467-018-03000-w)
Supplement: Supplementary file 1 — Supplementary Information [file 41467_2018_3000_MOESM1_ESM.pdf]

Supplementary Information for 'Spectroscopy of bulk and few-layer superconducting $NbSe_2$ with van der Waals tunnel junctions'

T. Dvir,[1] F. Massee,[2] L. Attias,[1] M. Khodas,[1] M. Aprili,[2] C. H. L. Quay,[2] and H. Steinberg[1]

[1] *The Racah Institute of Physics, the Hebrew University of Jerusalem, Israel*

[2] *Laboratoire de Physique des Solides (CNRS UMR 8502), Btiment 510, Universit Paris-Sud/Universit Paris-Saclay, 91405 Orsay, France*

(Dated: January 11, 2018)

Supplementary Note 1. THICKNESS AND STRUCTURE OF THE TUNNEL BARRIERS

The high optical contrast between layers of different thickness of transition metal dichalcogenides (TMDs) allows easy identification of the thickness of the tunnel barrier. Supplementary Figure 1 shows the optical image of the barrier on the PDMS immediately after it was exfoliated (panel c) and on top of the bulk $NbSe_2$ flake after the transfer procedure (panel b). We can see that the source electrode was deposited above a region of 4 and 5 layer thick MoS_2. As a result of exponential dependence of the tunnel current on the barrier thickness, only the 4 layer part of the junction is significant to the measurement. Hence we expect the effective junction area to be 1.6 μm^2 and the barrier thickness to be between 2.4 nm and 2.6 nm. In contrast to the optical imaging, AFM does not provide a reliable measure of height between two different materials and cannot measure the thickness of the barrier. However AFM reveals some structures which are probably due to PDMS residue from the transfer process (panel a). A cross-section of some of these features in the area of the junction shows height variation on the scale of 7 nm. The usual cleaning techniques of heat annealing cannot be used here due to the sensitivity of $NbSe_2$ to heat. The effect of this structure is most likely to reduce the effective area of the junction to the non-contaminated region. As discussed below, the effective area of the junction is of the same order of magnitude as the observed area, showing the robustness of this method to imperfections.

Supplementary Note 2. ESTIMATE OF THE BARRIER TRANSPARENCY

We can estimate the transparency of our tunnel barrier $\mathcal{T}$ from the well-known expression from Sharvin [1]:

$$G_{\rm N} = \frac{2e^2}{h}\frac{k_{\rm F}^2 A}{4\pi}\mathcal{T}, \tag{1}$$

where $G_{\rm N}$ is the junction conductance in the normal state, A is the area of the junction, $k_{\rm F}$ the Fermi momentum and $\mathcal{T}$ the average transmission of each conductance channel. In the bulk device, we measure $G_{\rm N} = 7\ \mu$S for $A = 1.6\ \mu$m^2. $k_{\rm F}$ in metals is usually $\sim 10^{10}$ m^{-1} and it is about half this value in $NbSe_2$. Taking the lower value, we get $\mathcal{T} \sim 3 \times 10^{-8}$.

We can make an independent estimate of $\mathcal{T}$ using the textbook WKB formula for a square

barrier of thickness d and height U [2]:

$$\mathcal{T} = \exp(-2d\sqrt{2m^*U}/\hbar) \tag{2}$$

where m^* is the effective mass of the electron in the barrier, here MoS_2.

The gap of few layer MoS_2 at the Γ point in the Brilloiun zone is on the order of 2 eV, whereas the effective mass is generally a fraction of 1. Taking $U = 1$eV, $m^* = m/2$ (m being the bare electron mass), and d in the range 2.4–2.6 nm we find $\mathcal{T} \sim 3 \times 10^{-8}$–$6.5 \times 10^{-9}$, consistent with the Sharvin estimate.

We can make a more rigorous estimate of U (and thus $\mathcal{T}$) by using Brinkman et al.'s result [3] for the conductance across a trapezoidal barrier with diffuse boundaries, together with measurements of the high bias conductance of our junction:

$$\frac{G(V)}{G(0)} = 1 - \frac{A_0 \Delta\phi}{16\bar{\phi}^{3/2}} eV + \frac{9}{128}\frac{A_0^2}{\bar{\phi}}(eV)^2 \tag{3}$$

where V is the voltage across the barrier, $\bar{\phi}$ is the mean barrier height, $\Delta\phi$ the barrier height difference on the two sides of the trapezoid, $\mathtt{d}$ the barrier width and $A_0 = 4\sqrt{2m^*}\mathtt{d}/3\hbar$. In these expressions, $\mathtt{d}$ is in units of Å, while $\bar{\phi}$, ϕ and V are in units of volts.

Far from the Fermi level, the conductance of our junction indeed rises (Supplementary Figure 2). This rise is not perfectly parabolic and is likely due, in part, to factors other than barrier transparency and asymmetry. Therefore, fitting a parabola to the background, i.e. assuming that the rise is due almost entirely to the barrier, will give us a worst-case scenario or minimum possible barrier height. From the fit to our data to Equation 3 using $\mathtt{d} = 20$Å, we find $\bar{\phi} \sim 0.8$ V, not so different from what we assumed previously. If we use this, and $d = 2.4$–2.6 nm as before, $\mathcal{T} \sim 2 \times 10^{-7}$–$5 \times 10^{-8}$.

Considering all of the above, $\mathcal{T}$ is likely in the 10^{-8} range or close to it.

Supplementary Note 3. FITTING THE SPECTRA OF ULTRATHIN $NbSe_2$ TO THE SSM MODEL

The two-band SSM can be used to fit the spectra obtained from 3- and 4- layers $NbSe_2$, in addition to the fit to the bulk sample discussed in the main text. The fits are shown in Supplementary Figure 3 and the fit parameters are given in Supplementary Table I. It

Thickness	Δ_1^0 [meV]	Δ_2^0 [meV]	Γ_1 [meV]	Γ_2 [meV]	N_1	N_2	T [K]
Bulk	1.26	0.29	0.27	1.25	1	0.18	0.44
4 layers	0.65	0.42	0.14	0.03	1	0.03	0.5
3 layers	0.42	0	0.09	0.09	1	0.04	0.32

Supplementary Table I: Summary of the fitting parameters to the SSM model

is clear that the value of Δ_1^0 decreases with a decreasing number of layers. In addition, the coupling constant Γ_1, which is associated with the rate of scattering of electrons from the band with the larger coupling, also decreases with decreasing number of layers. The values of Δ_2^0 and Γ_2, however, cannot be determined unambiguously. In fact, there is an anti-correlation between the possible values they can have. The values showed in Table I are the values used to generate the fits given here and in the main text.

The KZ model extends the SSM model to describe the spectrum under the application of in-plane magnetic field. This requires the introduction of a depairing parameter Γ_i^{AG} for each band. We show the value of Γ_i^{AG} extracted from the model fit for each device in the table below. We stress that the observed change in the spectrum with magnetic field is very small for the thin samples, and in the band with the larger gap of the bulk samples. Thus, the obtained values of Γ_i^{AG}should be treated as bounds, rather then accurate numbers. Further study, conducted at higher fields would allow for a more precise estimation.

The depairing represented by Γ_i^{AG} can originate from orbital depairing or from the interaction between the electrons' spin the applied magnetic field. Interpreting the depairing in terms of diffusive orbital effect allows us to extract the diffusion constant, D, as explained in the main text. Assuming a Fermi velocity $v_F = 10^5$ ms^{-1}, typical to $NbSe_2$ K bands, we can extract the elastic mean free path, l_{mfp} and the time between scatterings, τ. The mean free path was previously found to be in the range $l_{\mathrm{mfp}} \approx$ 30-80 nm [4, 5]. Table II show that this interpretation gives plausible values for all bands except the second band in the 3L device. The values for the second bands in the bulk and 4L device are slightly higher than expected. This can be resolved by assuming a higher Fermi velocity, consistent with their identification as originating from the Se derived Γ band. In the 3L device, the depairing is too high to originate from orbital depairing, and has to be associated with spin-field interaction.

To interpret the depairing in terms of the interaction between the field and the spins, we

Thickness	$\Gamma^{\mathrm{AG}}\left(\frac{H_{\parallel}}{1\,T}\right)^2$ [μev]	D [cm^2 sec^{-1}]	l_{mfp} [nm]	τ [fsec]	τ_{SO} [fsec]	Δ_{SO} [meV]
Bulk, band 1	50	3	11	0.1	-	-
Bulk, band 2	640	40	135	1.5	-	-
4 layers, band 1	0.8	6	18	0.2	0.08	8.2
4 layers, band 2	2	14	46	0.5	0.2	3.3
3 layers, band 1	0.5	6	20	0.2	0.05	13
3 layers, band 2	6	75	250	2.7	0.6	1.1

Supplementary Table II: Summary of the fitting parameters to the KZ model and their possible interpretations.

note that away from the extreme paramagnetic limit, we should observe Zeeman splitting of the quasiparticle peaks. The effect can be suppressed by the spin-orbit interaction in one of two ways - spin flip during scattering process, that averages the projection of the spin on the magnetic field; and renormalization of the in-plane magnetic field by the effective out-of-plane field generated by the Ising spin-orbit coupling. The former effect is quantified by a typical time for spin flip, τ_{SO}, whereas the latter is quantified by a spin orbit energy, Δ_{SO} [6]. Both enter the KZ model through Γ_i^{AG}. Table II shows that for the bands 1,2 of the 4 layer device and band 1 of the 3 layer device, τ_{SO} is shorter than the typical elastic scattering time, making this interpretation implausible.

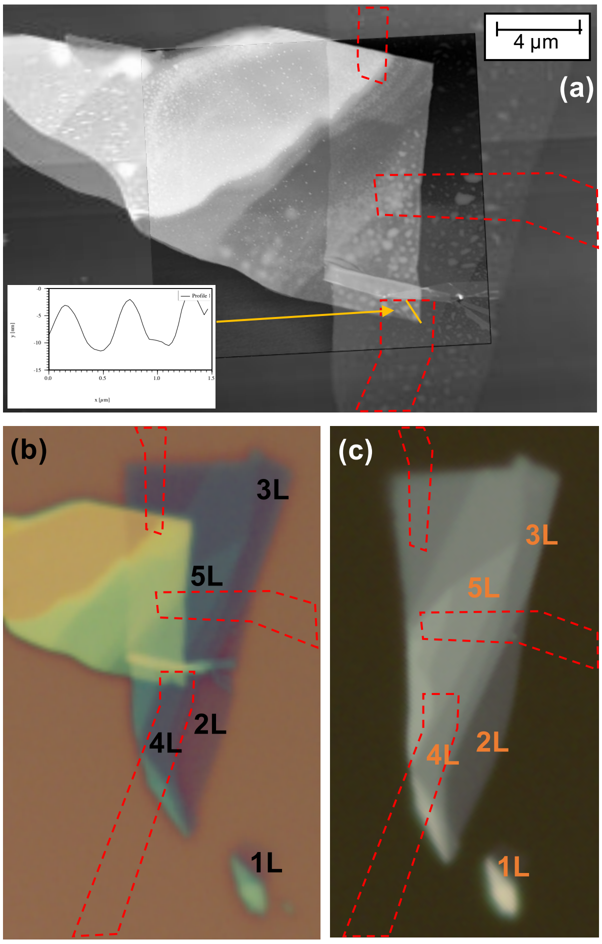

Supplementary Figure 1: **AFM and optical images of the device. a,** AFM imaging of the bulk device discussed in the main text. Position of the tunnel electrodes marked in dashed-red. Inset: cross section of the solid blue line, showing the typical size of the dirt on the device. **b,** optical image of the two flakes prior to the deposition of the electrodes. Black numbers mark the number of layers observed, from 1 to 5. **c,** optical image of the MoS_2 flake on the PDMS prior to the transfer process. Orange numbers mark the number of layers observed, from 1 to 5.

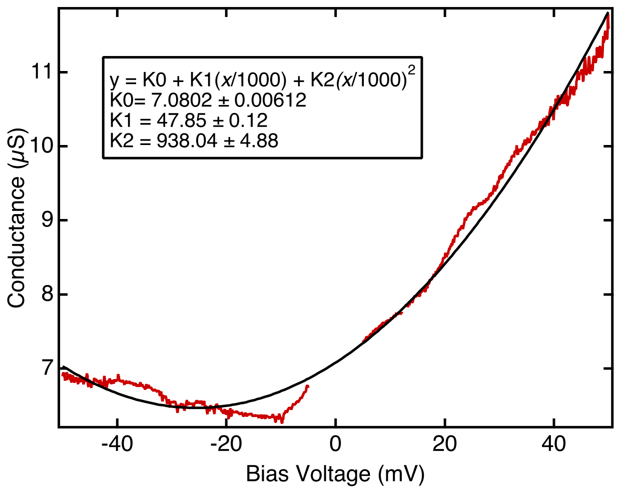

Supplementary Figure 2: **Conductance background.** Conductance as a function of voltage at energies above the superconducting gap (red) with a parabolic fit (black). The fit allows us to estimate our barrier height.

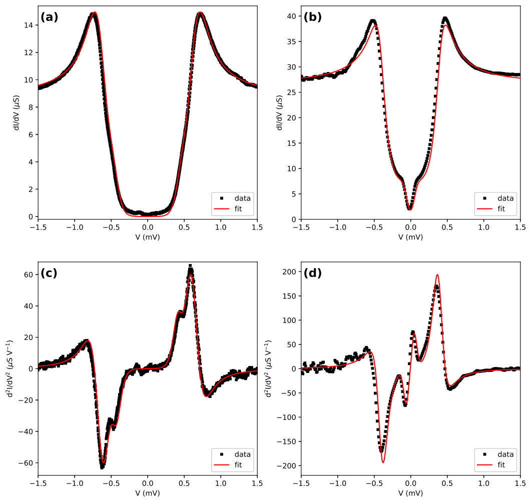

Supplementary Figure 3: **SSM model fits to 3- and 4-layer NbSe$_2$, a.** Differential conductance curve and (**c.**)the second derivative taken with the 4L sample and fits to SSM model. **b,** Differential conductance curve and (**d.**) the second derivative taken with the 3L sample and fits to SSM model. Fit parameters are given in table I

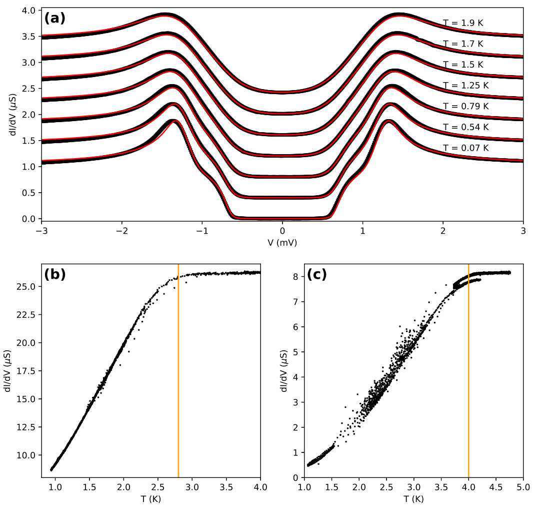

Supplementary Figure 4: **Temperature dependent differential conductance,** a. Differential conductance curves taken with the bulk sample and fits to SSM model. To fit the data, first the lowest temperature curve was fitted to the SSM model as discussed in the main text. Then, the other curves were fitted to the model with the same parameters, where only the temperature and order parameters (T, Δ_1, Δ_2) were allowed to change. Best fit was obtained when the order parameters remained unchanged. b. Zero bias conductance as a function of temperature for the three layer sample. This data was used to determine T_c, as transport data was unavailable. The critical temperature was defined by 5% reduction in the conductance.Orange line marks T_c. c. Same for the four layer sample.

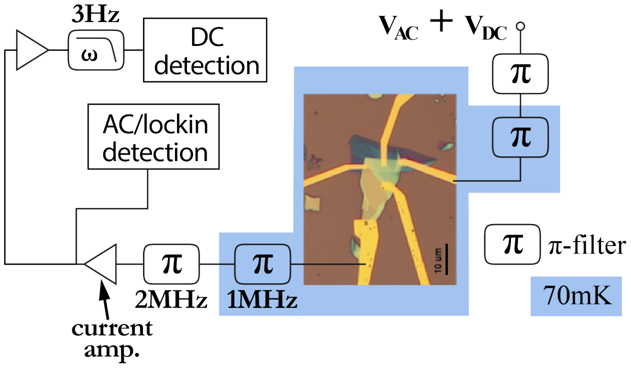

Supplementary Figure 5: Detailed diagram of the measurement circuit used in the experiment. All π-filters at low temperature have cutoff frequencies of 1MHz while those at room temperature have cutoff frequencies of 2MHz. The amplitude of the AC excitation V_{AC} is 15μV in all the figures of the main text. Measurements at lower V_{AC} showed that, between 2μV and 15μV, there was no discernible distortion of $G(V)$; the higher excitation voltage was thus chosen in order to have a better signal-to-noise ratio.

[1] Sharvin, Y. V. A possible method for studying Fermi surfaces. *JETP* **48**, 984–985 (1965).

[2] Griffiths, D. J. *Introduction to quantum mechanics* (Pearson Education India, 2005).

[3] Brinkman, W. F., Dynes, R. C. & Rowell, J. M. Tunneling Conductance of Asymmetrical Barriers. *Journal of Applied Physics* **41**, 1915 (1970).

[4] Renner, C., Kent, A. D., Niedermann, P., Fischer & Lévy, F. Scanning tunneling spectroscopy of a vortex core from the clean to the dirty limit. *Physical Review Letters* **67**, 1650–1652 (1991).

[5] Prober, D. E., Schwall, R. E. & Beasley, M. R. Upper critical fields and reduced dimensionality of the superconducting layered compounds. *Physical Review B* **21**, 2717–2733 (1980).

[6] Lu, J. M. *et al.* Evidence for two-dimensional Ising superconductivity in gated MoS2. *Science* **350**, 1353–1357 (2015).
